# PCR-based Diagnosis of *Toxoplasma* Parasite in Ocular Infections Having Clinical Indications of Toxoplasmosis

**Published:** 2017

**Authors:** Atieh FARHADI, Ali HANILOO, Asghar FAZAELI, Siamak MORADIAN, Mehdi FARHADI

**Affiliations:** 1. Dept. of Parasitology and Mycology, School of Medicine, Zanjan University of Medical Sciences, Zanjan, Iran; 2. Fellowship in Vitreoretinal Surgery, Ophthalmic Research Center, Labbafinejad Medical Center, Shahid Beheshti University of Medical Sciences, Tehran, Iran

**Keywords:** Ocular toxoplasmosis, Nested-PCR, Aqueous, Vitreous, Iran

## Abstract

**Background:**

The diagnosis of ocular toxoplasmosis is mainly based on clinical features. However, ocular fluid testing by PCR may be very helpful for approval or rejection of this etiology. In this study, we utilized a nested-PCR technique, targeting the B1 partial sequence to analyze the aqueous and vitreous samples for evaluating the presence of the *Toxoplasma* DNA.

**Methods:**

Fifty aqueous or vitreous humor samples were obtained from patients with clinical features of ocular toxoplasmosis admitted to ophthalmology hospitals and clinics in Iran, within 2014. The samples were subsequently subjected to DNA extraction and purification. For nested amplification of the *Toxoplasma* B1 gene, two primer pairs were used. The outer and inner primers are expected to produce a 193 bp and a 96 bp fragments, respectively.

**Results:**

The first-round PCR resulted in the detection of *T. gondii* in 58% of samples by amplification of the expected 193bp DNA fragment. The nested-PCR using the inner primers, detected 15 additional samples from those with negative amplicons in the first round PCR (overall positivity of 88%). In addition, vitreous samples showed relatively more positive cases than aqueous humor in detection of the infection.

**Conclusion:**

The nested-PCR protocol using the B1 gene, with the high detection power, could be a useful complimentary method to clinical diagnose of ocular toxoplasmosis.

## Introduction

*Toxoplasma gondii* is a protozoan parasite that can affect many species of warm-blooded animals, including human. Based on the serological studies, about one-third of the world populations have been exposed to *Toxoplasma* parasite (1, 2). Although *Toxoplasma* infection is often mild and subclinical, it can have severe consequences in particular groups. *Toxoplasma* tachyzoits pass through the placenta and cause congenital infection with the central nervous system and ocular involvement. In addition, acute or reactivated chronic infections in patients with impaired immune system, especially those with AIDS, have the most clinical outcomes (2).

*T. gondii* is one of the most important parasites responsible for uveitis and retinochoroiditis. In fact, ocular toxoplasmosis may be the most common type of posterior uveitis worldwide (3–4). Similar status has been reported in Iran (5–6). In a study on 329 patients with uveitis referred to Farabi Tertiary Eye Care Center in Isfahan, Iran (6), toxoplasmosis was the most common cause of posterior uveitis (93.5%) and the most common known etiology of uveitis (22.2%). The incidence of ocular toxoplasmosis is usually in twenties through forties, following congenital infections (7) and this form of the disease, is rarely seen in patients with acute acquired toxoplasmosis.

Diagnosis of ocular toxoplasmosis is routinely based on clinical findings. The typical signs of infection consists necrotizing retinitis or retinichoroiditis near to a variably pigmented chorioretinal scar (3). However, in the case of atypical toxoplasmosis that is indistinguishable from other infectious and non– infectious retinitis, ocular fluid testing by PCR may be very helpful for approval or rejection of this etiology. Serological tests for the diagnosis of ocular toxoplasmosis alone are of little value. IgG positive titer with typical lesions, commonly leads to diagnosis of ocular toxoplasmosis, and a negative serology of anti-toxoplasma antibodies, rule out *Toxoplasma* as etiologic agent of posterior uveitis (3).

In recent decade, molecular methods as sensitive techniques have been widely used for the diagnosis of toxoplasmosis (8–13). Molecular diagnosis of *Toxoplasma* is commonly based on identifying specific DNA sequences of parasite in biological and clinical samples, using highly conserved regions such as the B1 gene with 35 copies, 529 bp (AF146527) element with 200–300 copies, and ITS-1 (internal transcribed spacer 1) with 110 copies in the genome (8). The sensitivity of the ITS-1 and 18S rDNA fragments in the diagnosis of *T. gondii*, has been reported to be similar to the B1 gene (10, 11). Although, in the initial study, the 529 bp element showed a sensitivity of 10 to 100 times higher than the B1 gene (12), in later studies, the whole or part of the 529 bp fragment was deleted or mutated in some strains of the parasite, which could lead to false-negative results in some samples (13, 14). The B1 gene sequence conserved in almost all strains of *T. gondii*, showed better performance in diagnosis of *Toxoplasma* infection (10, 15). Several cases of ocular disorders usually referred to different eye care centers and clinics are categorized as ocular toxoplasmosis solely based on clinical features and receives consequently anti-*Toxoplasma* treatments.

In this study, we utilized a nested-PCR technique, targeting the B1 partial sequence to analyze these clinical samples, for evaluating the presence or absence of the *Toxoplasma* parasite DNA.

## Materials and Methods

### Sampling

The Institutional Research Ethics Committee of Zanjan University of Medical Sciences approved this study (Ref No. ZUMS.REC.1392.45), and informed consent was obtained from all patients before intraocular paracentesis. Considering ethical issues, aqueous and vitreous samples were collected from patients scheduled for inevitable injection of intravitreal clyndamycin, including patients who did not accept systemic anti toxoplasmosis therapy and cases with systemic treatment contraindications. Fifty ocular fluid samples (aqueous humor or vitreous humor) were obtained from patients with clinical features of ocular toxoplasmosis admitted to ophthalmology hospitals and clinics in Tehran, including Farabi Hospital, Labbafinejad Hospital, Hazrat Rasoul Hospital, Vanak Eye Center, Bina Eye Hospital (32 samples), and ophthalmology centers and hospitals in Isfahan, Bandar Abbas, Sari, Gorgan, Mashhad and Rasht, Iran (18 samples) in 2014. The diagnosis was made according to the clinical features and symptoms consistent with ocular toxoplasmosis by ophthalmologist. Samples of the patients’ ocular fluids in approximate volumes of 150 μl were taken in sterile conditions in the operating rooms and stored at −20 °C until analysis.

### DNA extraction and PCR

The ocular fluid samples were centrifuged for 10 min at 3000 *g*. The pellets were washed twice in PBS and were incubated for 5 min at 100 °C in 50 μl of deionized distilled water containing 20 μg/ml of RNase. The samples were subsequently subjected to DNA extraction; a sample of *Toxoplasma* RH strain tachyzoites as a positive control was used along with the clinical samples. The washed pellets were incubated at 56 °C for 2 h in the lysis buffer containing 100 μg/ml proteinase K, 50 mM Tris-HCl (pH 8.0), 25 mM EDTA, and 2% SDS (16). Then, DNA purification was performed by phenol–chloroform-isoamylalcohol method, precipitated with isopropanol and washed with 70% ethanol as described elsewhere (17).

For PCR amplification of the *Toxoplasma* B1 gene, two primer pairs (9) were used. They included one pair of outer primers (sense: 5′-GGA ACT GCA TCC GTT CAT GAG-3′, antisense: 5′-TCT TTA AAG CGT TCG TGG TC-3′), and one pair of inner primers (sense: 5′-TGC ATA GGT TGC AGT CAC TG-3′, antisense: 5′-GGC GAC CAA TCT GCG AAT ACA CC-3′). The outer primers and inner primers are expected to produce a 193 bp and a 96 bp fragments, respectively.

### First-round amplification

Each 25 μl PCR mixture contained 250 μM each dNTP, 10 mM Tris–HCl (pH 8.3), 50 mM KCl, 1.5 mM MgCl_2_, 10 pmol of each outer primers, 1 U of *Taq* DNA polymerase and approximately 100 ng extracted DNA samples. PCR amplification was started with an initial denaturation at 94 °C for 4 min, followed by 36 cycles containing denaturation at 94 °C, annealing at 57°C, and extension at 72°C, each step for 45 sec. It was completed with a final extension for 10 min at 72 °C in a thermo cycler (CORBETT Research, Australia). A positive control using DNA from tachyzoite of *Toxoplasma* RH strain and a negative control using sterile water were included into the PCR reactions. The PCR products were analyzed by electrophoresis in 2% agarose gels stained with safe stain. The amplicons were visualized and recorded digitally by a gel documentation system (UVdoc., UK).

### Nested amplification

The negative products of the first-round amplification along with negative and positive controls were further investigated by nested-PCR using the second (inner) primer pair. All conditions and PCR mixture was the same as the first-round amplification, except for different annealing temperature of 62 °C and the DNA template that was the first-round products.

## Results

The majority of the patients were in the age groups of 30–39 (38%) and 20–29 yr (30%), with the mean of 36.1±9.0 (ranged 23 to 59) yr. Most of the patients (70%) were female. Other detailed demographic data are presented in Table 1.

**Table 1:** Demographic data of patients with ocular toxoplasmosis

**Age group (yr)**	**No. (%)**	**Gender**		**Affected eye**		**Sample type**		**Residence**	
		**Male**	**Female**	**Right**	**Left**	**Aqueous**	**Vitreous**	**Tehran**	**Other cities**
20–29	15 (30)	3	12	3	12	8	7	8	7
30–39	19 (38)	6	13	10	9	9	10	13	6
40–49	12 (24)	6	6	11	1	7	5	9	3
50–60	4 (8)	0	4	2	2	4	0	2	2
Total	50 (100)	15 (30)	35 (70)	26 (52)	24 (48)	28 (56)	22 (44)	32 (64)	18 (36)

**Fig. 1:**
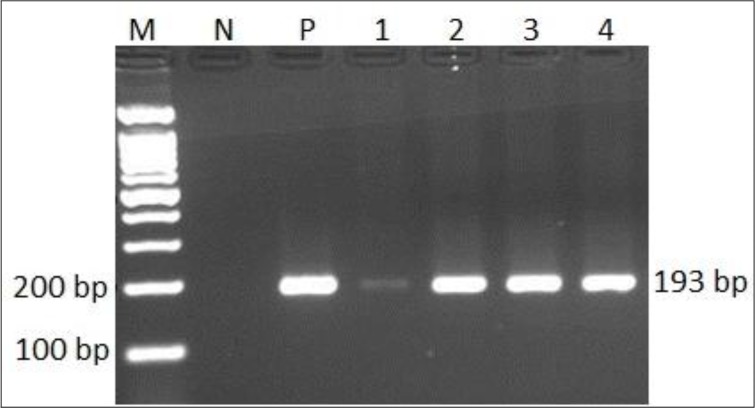
The first round PCR products of 193 bp *Toxoplasma* B1 gene using ocular fluid samples of patients resolved on 2% agarose gel. M: molecular size marker (100 bp ladder); N: negative control (no DNA); P: positive control (*T. gondii* RH strain DNA) and lanes 1–4: patiens samples.

**Fig. 2:**
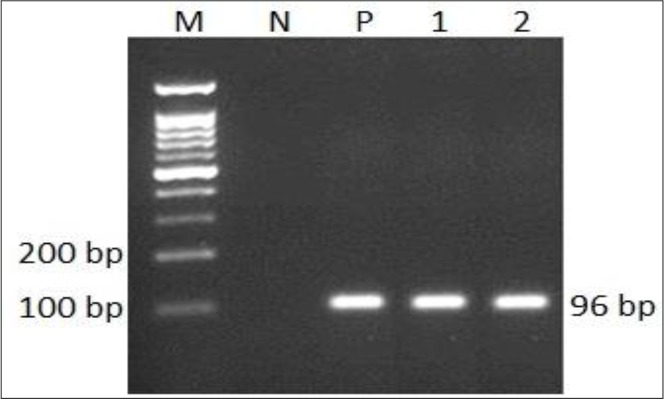
The Nested-PCR products of 96 bp *Toxoplasma* B1 gen from ocular fluid samples of patients resolved on 2% agarose gel. M: molecular size marker (100 bp ladder); N: negative control (no DNA); P: positive control (*T. gondii* RH strain DNA) and lanes 1–2: positive samples.

The first-round PCR using the outer primers resulted in the detection of *T. gondii* parasite in 29 out of 50 samples (58%) by amplification of the expected 193 bp DNA fragment (Fig. 1). The nested-PCR using the inner primers, detected 15 additional samples from those with negative amplicons in the first round PCR (Table 2, Fig. 2). In fact, the nested PCR increased the sensitivity of this method for the diagnosis of ocular toxoplasmosis. Although, the sensitivity of nested-PCR according to sample type, aqueous or vitreous, was not statistically different, but vitreous yield more relative positive cases (Table 3).

**Table 2:** Detection of *T. gondii* by nested-PCR in ocular infections having clinical indications of toxoplasmosis

**PCR**	**No.**	**Positive (%)**	**Negative**
First round	50	29 (58)	21
Nested	21	15 (71)	6
Overall	50	44 (88)	6

**Table 3:** Sensitivity of nested-PCR in detection of ocular toxoplasmosis in two sample types (aqueous and vitreous)

**Nested-PCR**	**Sample type (percent)**		**Total**
	**Aqueous**	**Vitreous**	**(Percent)**
Positive	22 (81.5)*	22 (95.7)*	44 (88.0)
Negative	5	1	6
Total	27	23	50

* *P*-value was greater than 0.05 by exact fisher test.

## Discussion

In this study, 50 patients with clinical diagnosis of ocular toxoplasmosis were enrolled. The most patients were in the third or fourth decade of their life that was consistent with the age distribution of the most studies (e.g. 3, 7). However, in some studies, the patients were younger than our study patients were. For example, the average age of patients with ocular toxoplasmosis was 25.1 and 24.3 years in the studies of Isfahan (6), and Tehran (18), respectively. Our patients consisted of 35 (70%) female and 15 (30%) males, with similar sex distribution to other studies (3, 6, 7, 18).

Apart from atypical cases, recognition of ocular toxoplasmosis is based on clinical findings (3). In addition, parasite-specific DNA is detectable in ocular fluids of these patients using different techniques of PCR. Whereas, these methods have often-high sensitivity in the detection of parasite DNA, the standard test has not been introduced for the diagnosis of ocular toxoplasmosis in ocular fluids (19). The B1 gene with conserved sequences in almost all *T. gondii* strains, have been used for the detection of parasite in various clinical samples including ocular fluids, and reported to have acceptable sensitivity and specificity (8, 10, 15).

In our study, although the first round PCR of the B1 target in ocular fluids was not highly sensitive (with 58% detection rate), adding the second round amplification by the nested primers, considerably increased the sensitivity of this PCR technique up to 88%. This finding is consistent with many published data. Other researchers concluded that the B1 gene would be preferred for the diagnosis of *T. gondii* in ocular samples, when compared to other targets, i.e. P30 and rDNA studied by Jones *et al.* (10), and 592-bp (AF146527) and rDNA that was reported by Okay et al. (15). They also reported that, the B1 gene PCR, preferably nested amplification is far more sensitive than the other two PCRs in detection of *Toxoplasma* in amniotic samples. In a study in France (20) by real-time PCR targeting the 529-bp element for the diagnosis of toxoplasmic retinochoroiditis in patients with atypical uveitis, parasite DNA was detected in 55% of patients. In another French study (21) with the same method and DNA target, the positive rate was even lower (38.2% of the 34 samples). While, in our study the B1 nested PCR could detect the parasite DNA in 88% of the samples. In the latter French study, the sample volume used for DNA extraction was unusually small (10 μl), which may have been effective in lowering the sensitivity of their PCR reaction. Although, theoretically, the more repetitive DNA sequences could lead to higher sensitivity, only a few studies confirmed this theory on detection of *Toxoplasma* DNA. The 529 bp fragments with 200–300 repeats were identified in the *Toxoplasma* genome and this target was more sensitive than the B1 gene in amplification systems (12). However, in some strains of the parasite, whole or part of the 529 bp fragment is deleted or mutated and could lead to false-negative results in some samples (13, 14).

Our samples consisted of 23 vitreous and 27 aqueous humor. The PCR protocol on vitreous samples, comparing to the aqueous humor, showed a higher sensitivity in diagnosis of ocular toxoplasmosis, (95.7% vs 81.5%). Considering that the vitreous fluid is in close contact with the necrotic lesions, the parasite particles could enter the vitreous more easily than the aqueous humor. Analysis of vitreous fluid by PCR is valuable for tracing of *Toxoplosma*, particularly in atypical infections. Seven out of 15 patients with atypical toxoplasmosis, vitreous fluid examination was positive for the presence of *T. gondii* DNA by PCR (22). In a recent study on a series of patients with posterior infectious uveitis, Harper and colleagues have reported the overall sensitivity of 67% for PCR in detection of ocular toxoplasmosis, considering the clinical criteria as the gold standard; but they found positive cases with similar rate in both aqueous humor and vitreous were analyzed (23). Despite the higher sensitivity of vitreous analysis, vitreous tap is recommended only in severe atypical or complicated cases and aqueous tap poses relatively less risk to the eye (3). Another factor that could affect the sensitivity of molecular techniques in the diagnosis of ocular toxoplasmosis is the status of the patient′s immune system. Immunocompetent individuals were reviewed; *Toxoplasma* DNA could be detected within aqueous samples in utmost 30%–40% of the clinically diagnosed patients (19). In immunocompromised patients, but toxoplasma DNA was detectable in 75% of the clinically diagnosed cases; no reason was presented in description of the sensitivity differences between two groups of patients with different immune system conditions, and it seems to need further investigation.

## Conclusion

The nested-PCR protocol using the B1 gene appears to be highly sensitive in detection of *T. gondii* DNA in ocular fluids of the patients affected by ocular toxoplasmosis. In addition, vitreous sample yields relatively more positive cases than aqueous humor in detection of the infection. The PCR protocol used in this study could be a useful complimentary method to clinical diagnose of ocular toxoplasmosis.
